# Supplementary material for: Determinants of modern contraceptive use among married and sexually active unmarried women aged 15–49 years old in Cambodia: How are geographical and socio-demographic factors associated with access?
Source: PLOS Glob Public Health. 2025 Sep 2;5(9):e0004346. doi: 10.1371/journal.pgph.0004346 (PMC12404472; doi:10.1371/journal.pgph.0004346)
Supplement: Attachment — Submitted filename: PLOS_Reviewer_Comment.docx [file pgph.0004346.s002.docx]

# Formal Reviewer Comment for PLOS Global Public Health

Manuscript ID**: PGPH-D-25-00309

**Title: Determinants of Modern Contraceptive Use among Women of Reproductive Age in Cambodia: How Are Geographical and Socio-Demographic Factors Associated with Access?**

# Reviewer’s Assessment

## Summary of the Manuscript

The manuscript presents an important and well-structured study on modern contraceptive use in Cambodia, using nationally representative data from the Cambodia Demographic and Health Survey (CDHS) 2021–2022. The study highlights key socio-demographic and geographic determinants influencing contraceptive use, providing valuable insights for public health interventions. The mixed statistical approach, including descriptive and logistic regression analyses, enhances the study’s rigor.

While the research is well-executed and relevant to global public health discussions, some areas require clarification and refinement to improve transparency, methodological rigor, and the clarity of interpretations. Below, I outline specific recommendations for improvement.

## Major Comments

1. Study Design & Justification-

- The manuscript does not explicitly state that it is a *cross-sectional* study in the methodology section. Clarifying this is essential to set appropriate expectations regarding causality.
- The rationale for using this dataset should be expanded upon. How does it build on or differentiate itself from prior research using CDHS data?

2. Methodological Transparency

- Sample Size Justification: It is unclear whether a *power calculation* was conducted to determine the adequacy of the sample size. If not, please explain how the current sample size is sufficient.
- Selection Bias: The study focuses only on married and sexually active women, excluding unmarried and inactive women. While this is logical, the paper should explicitly *acknowledge this limitation*.
- Confounder Control: More information is needed on how *confounders were selected* for adjustment in the regression models. Was a *multicollinearity check* conducted?

3. Statistical Analysis & Interpretation

- The finding that *motorcycle ownership significantly increases contraceptive use*(AOR = 1.29, 95% CI: 1.09–1.53) is important. The authors should emphasize how *mobility constraints* affect healthcare access in rural areas.
- The paper should explain *why women living further from healthcare facilities showed higher contraceptive use*, as this contradicts expected trends. This counterintuitive result should be further analyzed could it be due to self-selection bias, where women highly motivated to use contraception travel longer distances?
- The study finds that contraceptive use **increases with parity** (AOR for women with three or more children = 30.76). This suggests that **family planning efforts are more effective after women have had multiple children**, but strategies to promote earlier adoption should be discussed.
- A *subgroup analysis* (e.g., wealthier vs. poorer women, urban vs. rural) would strengthen the findings by identifying variations in contraceptive access.

4. Discussion & Policy Implications

- The *novelty of the study* should be clearly stated. What is new about these findings compared to prior studies?
- The *policy implications *need to be more concrete. Instead of general recommendations, can the authors suggest *specific interventions* (e.g., mobile health clinics, targeted subsidy programs)?
- The discussion should better integrate findings from *other Southeast Asian countries* to contextualize Cambodia’s contraceptive trends.
- Expand the discussion on age-related patterns**:
   -Why do older women use contraception less? Is it fertility-related, preference for traditional methods, or reduced health-seeking behavior?
- Provide clearer policy recommendations for urban areas**, where contraceptive use is unexpectedly lower.

5. Limitations & Ethical Considerations

- The *limitations section* should explicitly mention *self-reported data bias*, potential *causality issues due to the cross-sectional nature*, and *unmeasured confounders* such as cultural norms.
- Ethical considerations are well-addressed, but **more details on informed consent** during CDHS data collection would strengthen the manuscript’s transparency.

## Minor Comments

## The abstract should explicitly state the *study design* (cross-sectional).

- Tables should be *formatted for readability*, particularly those with dense numerical data.
- There are some *minor grammatical issues* a language edit would improve clarity.
- Improve variable categorization explanations* (e.g., why specific cutoff points for distance to healthcare were chosen).
- Discuss dual-method contraceptive use (modern + traditional), if relevant.

## Recommendation

** Accept with Major Revisions and Minor Revisions** – The study is methodologically sound and contributes to public health knowledge, but revisions are needed to **clarify methodology, enhance result interpretation, and strengthen policy implications**.

I appreciate the authors' effort in conducting this research and look forward to seeing an improved version of the manuscript.
