# Supplementary material for: Determinants of modern contraceptive use among married and sexually active unmarried women aged 15–49 years old in Cambodia: How are geographical and socio-demographic factors associated with access?
Source: PLOS Glob Public Health. 2025 Sep 2;5(9):e0004346. doi: 10.1371/journal.pgph.0004346 (PMC12404472; doi:10.1371/journal.pgph.0004346)
Supplement: Attachment — Submitted filename: Response to Reviewers.docx [file pgph.0004346.s003.docx]

**Authors’ point-by-point responses to the editor and reviewers**

PGPH-D-25-00309

Determinants of Modern Contraceptive Use among Women of reproductive age in Cambodia: How are geographical and socio-demographic factors associated with access?

PLOS Global Public Health

**Dear editor and reviewers,**

We would like to thank all the reviewers and the editor for providing their valuable time and effort in reviewing our manuscript. We have addressed all comments where relevant, as listed below:

**5. Review Comments to the Author**

Please use the space provided to explain your answers to the questions above. You may also include additional comments for the author, including concerns about dual publication, research ethics, or publication ethics. (Please upload your review as an attachment if it exceeds 20,000 characters)

**Reviewer #1**: The paper is generally well-written with a good discussion. However, there are several definitions and analysis steps (including the variable selection and model comparison/evaluation) which need to be clarified. The authors should also suggest reasons for the differing relationships inferred from the chi-squared tests, univariate and multivariate logistic regression.

Thank you. Your review has definitely helped improve the quality of our paper further.

**Comments:**

**Reviewer #1**

• p.4, lines 108-113: These results from a past study using CDHS 2014 data can be mentioned again in the Discussion since they are similar to what is observed in terms of age, rural-urban and education factors for modern contraceptive use in this study.

**Author's response**: We agree, we have revised the Discussion section to reintroduce the findings from the previous CDHS 2014 study to highlight their consistency with our results (Page 18, Lines 423–425, and Page 21, Lines 460–464).

• p.4, lines 115-119: This is a bit confusing. Is there a distinction made between “access” and “use”? What are considered “general factors”? Are age, education and rural/urban factors included? It would be good to clarify how this study is different from [4] as well.

**Author's response:** We have clarified the distinction between “access” (geographic and socioeconomic determinants) and “use” (actual adoption of contraceptive methods). We have also highlighted how our study differs by focusing on specific access-related factors not extensively explored in previous CDHS analyses (Page 5, Lines 122–127).

• p.6, line 167-169: Could you clarify how the distance/time to reach the nearest health facility from home was computed? Were the survey participants first asked how they travel to the nearest health facility and then the distance/time calculated based on their chosen mode of travel?

**Author's response:** We have clarified that the CDHS 2021–2022 questionnaire directly asked women about the mode of transportation and their self-estimated travel time to the nearest health facility (Page 7, Lines 196–197).

• p.6, line 179: Define “DHS”. Please elaborate more on the principal components analysis. Did you use the first principal component as a wealth index and used quantiles to define the categories, e.g. poorest to richest?

**Author's response:** We have defined DHS (Demographic and Health Surveys) and expanded the description of the wealth index calculation through principal components analysis, using the first component and dividing it into quintiles (Page 7, Lines 208–217).

• p.8, line 234: What kind of media was considered?

**Authors’ response:** For clarity, the DHS survey collected women's exposure to any type of media, such as “Frequency of reading newspapers or magazines,” “Frequency of listening to radio,” and “Frequency of watching television”. We have now revised the manuscript accordingly (Page 7, Lines 219–223).

• p.12, line 279-281: Is the full set of variables considered in Table 2? How were the variables selected in the final multivariate logistic regression model? Please provide a goodness-of-fit measure and explain how the final model was obtained.

**Authors’ response:** We have explained that variables were selected based on a p-value ≤ 0.10 in bivariate analyses or based on theoretical relevance. We have reported model goodness-of-fit using the Hosmer-Lemeshow test and model discrimination using AUC (Page 9, Lines 252–254 and Page 15, Lines 333–335).

• p.12, line 288-296: Is the distance away from healthcare facilities confounded with the urban/rural variable? Why does the lower odds of women aged 35-49 of using modern contraception compared to adolescents aged 15-19 years conflict with the positive association of age with use in the chi-squared tests and unadjusted logistic regression on p.10? Is age confounded with e.g. parity? It would be important to interpret the adjusted odds ratios by holding all the other variables in the model fixed. On a related note, the insignificance of education and wealth in the multivariate logistic regression suggests that their effects on use could be explained by other covariates in the model.

**Authors’ response:**

A. We conducted multicollinearity diagnostics, and VIF values indicated no serious multicollinearity between distance to healthcare and rural/urban residence.
B. Regarding the differing results, we note that age and parity are correlated. After adjusting for parity, the initially positive association between age and contraceptive use weakened, suggesting confounding.
C. We have expanded the Discussion to explain these findings (Page 18, Lines 430–435).

• Figure 2: How are the probabilities calculated? Did you fix the values of the other covariates in the model? It would be good to describe the interpretation of the figure more. Conditional on parity, it seems that the difference in probability of use for women who own motorcycles or not is not significant in urban areas and only significant in rural areas for women who have 1-3 children.

**Authors’ response:** We used STATA's margins command to calculate predicted probabilities, holding other covariates at their mean or reference values. The figure explores a three-way interaction between motorcycle ownership, place of residence, and parity. We have elaborated this in the revised figure caption and Results section (Page 16, Lines 365–369).

• p.14, lines 317-318: In the multivariate logistic regression, living in a rural area is associated with higher use of modern contraceptives. This conflicts with the interpretation of the trends in Figure 2 when only 4 variables are examined.

• p.14, lines 320-321: Can you test for the “strong effect” of motorcycle ownership in rural settings in the multivariate logistic regression using an interaction term between the two variables?

**Authors’ response:** We have clarified that Figure 2 visualizes interactions conditional on parity, motorcycle ownership, and residence. We tested the interaction term between motorcycle ownership and residence, which was significant only in rural areas. We updated the Discussion accordingly (Page 18, Lines 436–439).

• p.16, lines 379-382: Could you check these inferences further by for example, examining the relationship between parity and wealth in the data?

**Authors’ response:** We found a correlation of -0.1194 between parity and wealth, indicating a weak, negative relationship. Also, we conducted multicollinearity diagnostics, and VIF values indicated no serious multicollinearity between parity and wealth status.

**Reviewer #2**

Minor comments:

• p.2, line 39-40 (Abstract, Results): The AORs are missing the 95% CIs.

**Authors’ response:** We have revised the abstract’s manuscript accordingly (Page 2, Line 40-42)

• p.2, line 53-54: Please add a reference or elaborate on how modern contraceptive methods may contribute to economic growth.

**Authors’ response:** Thank you for this suggestion. Following the journal's guidelines, references are not included in the Abstract. However, we have added a supporting reference and elaborated on this point in the Introduction section of the main text.

• p.3, line 99: “odd” is missing a “s”.

• p.4, line 130: What does “IR” stand for?

• p.5, line 166: There is a missing “a” for “Coastal”.

• p.9, line 249: There is a missing space between “95%” and “CI”.

• p.12, line 289: The p-value is missing.

• p.15, line 351: Please add the p-value and/or 95% CI to the AORs for geographic accessibility.

**Authors’ response:**

We apologize for the spelling, typographical and other mistakes. Kindly note that we have:

- Added missing “s” in “odds”.
- Defined “IR” (Individual Recode).
- Corrected missing “a” for "Coastal".
- Fixed formatting: added missing spaces near “95% CI” and p-values (throughout).

**Reviewer #2: Formal Reviewer Comment for PLOS Global Public Health**

Manuscript ID**: PGPH-D-25-00309

**Title: Determinants of Modern Contraceptive Use among Women of Reproductive Age in Cambodia: How Are Geographical and Socio-Demographic Factors Associated with Access?**

# Reviewer’s Assessment

## Summary of the Manuscript

The manuscript presents an important and well-structured study on modern contraceptive use in Cambodia, using nationally representative data from the Cambodia Demographic and Health Survey (CDHS) 2021–2022. The study highlights key socio-demographic and geographic determinants influencing contraceptive use, providing valuable insights for public health interventions. The mixed statistical approach, including descriptive and logistic regression analyses, enhances the study’s rigor.

While the research is well-executed and relevant to global public health discussions, some areas require clarification and refinement to improve transparency, methodological rigor, and the clarity of interpretations. Below, I outline specific recommendations for improvement.

## Major Comments

1. Study Design & Justification-

- The manuscript does not explicitly state that it is a *cross-sectional* study in the methodology section. Clarifying this is essential to set appropriate expectations regarding causality.
- The rationale for using this dataset should be expanded upon. How does it build on or differentiate itself from prior research using CDHS data?

**Authors’ response:**

We have stated clearly in the Methods that this is a cross-sectional study (Page 5, Lines 139–146). We have also elaborated that using CDHS 2021–2022 provides the most updated evidence on modern contraceptive use, focusing on geographical and socio-demographic determinants not extensively explored before (Page 5, Lines 122–127).

2. Methodological Transparency

- Sample Size Justification: It is unclear whether a *power calculation* was conducted to determine the adequacy of the sample size. If not, please explain how the current sample size is sufficient.

**Authors’ response:** No formal power calculation was conducted; however, the sample size exceeds DHS recommendations for national analyses.

- Selection Bias: The study focuses only on married and sexually active women, excluding unmarried and inactive women. While this is logical, the paper should explicitly *acknowledge this limitation*.

**Authors’ response:** We have clarified in the Study Population section that the exclusion of never-married or sexually inactive women does not affect our findings. Specifically, unmarried sexually inactive women—defined using the CDHS variable v528 (time since last sexual intercourse)—as those not currently married or in a consensual union (i.e., single, divorced, widowed, or separated) who reported having had sexual intercourse at least once in their lifetime but not within the 30 days preceding the survey—were excluded from the analysis (Page 6, Lines 167–174).."(Page 6, Lines 167–174).

- Confounder Control: More information is needed on how *confounders were selected* for adjustment in the regression models. Was a *multicollinearity check* conducted?

**Authors’ response:** Confounders were selected based on literature and from a bivariate Chi-Square test (Table 2), at significant associations (p ≤ 0.10); multicollinearity was assessed using VIFs (Page 8, Lines 249–252). Result reported in (Page 15, Lines 333-334).

3. Statistical Analysis & Interpretation

- The finding that *motorcycle ownership significantly increases contraceptive use*(AOR = 1.29, 95% CI: 1.09–1.53) is important. The authors should emphasize how *mobility constraints* affect healthcare access in rural areas.

**Authors’ response:** We have emphasized how motorcycle ownership improves healthcare access in rural areas in the discussion section (Page 21, Lines 449-456)

- The paper should explain *why women living further from healthcare facilities showed higher contraceptive use*, as this contradicts expected trends. This counterintuitive result should be further analyzed could it be due to self-selection bias, where women highly motivated to use contraception travel longer distances?

**Authors’ response:** We have discussed potential self-selection bias explaining why women farther from healthcare facilities may use more contraception (Page 23, Lines 512-521).

- The study finds that contraceptive use **increases with parity** (AOR for women with three or more children = 30.76). This suggests that **family planning efforts are more effective after women have had multiple children**, but strategies to promote earlier adoption should be discussed.

**Authors’ response:** We discussed how parity strongly influences contraceptive use, suggesting that interventions promoting earlier adoption are needed.

- A *subgroup analysis* (e.g., wealthier vs. poorer women, urban vs. rural) would strengthen the findings by identifying variations in contraceptive access.

**Authors’ response:** A subgroup analysis stratified by rural/urban residence has been partially incorporated in the marginal probability plots (**Fig. 3** and **Fig. 4**).

4. Discussion & Policy Implications

- The *novelty of the study* should be clearly stated. What is new about these findings compared to prior studies?

**Authors’ response:** We now state that this study uniquely examines access-related factors using the latest CDHS 2021–22 data (Page 5, Lines 122–127).

- The *policy implications *need to be more concrete. Instead of general recommendations, can the authors suggest *specific interventions* (e.g., mobile health clinics, targeted subsidy programs)?

**Authors’ response:** We have proposed specific policy recommendations such as mobile clinics and transportation support programs (Page 19, Lines 460–463).

- The discussion should better integrate findings from *other Southeast Asian countries* to contextualize Cambodia’s contraceptive trends.

**Authors’ response:** We contextualized our findings with evidence from other Southeast Asian countries (Page 18, Lines 421–423).

- Expand the discussion on age-related patterns**:
   -Why do older women use contraception less? Is it fertility-related, preference for traditional methods, or reduced health-seeking behavior?

**Authors’ response:** We expanded the explanation for lower contraceptive use among older women (Page 18, Lines 432–434).

- Provide clearer policy recommendations for urban areas**, where contraceptive use is unexpectedly lower.
- **Authors’ response: We have provided** (Page 22, Lines 449-502).

5. Limitations & Ethical Considerations

- The *limitations section* should explicitly mention *self-reported data bias*, potential *causality issues due to the cross-sectional nature*, and *unmeasured confounders* such as cultural norms.

**Authors’ response:** We expanded the Limitations section to acknowledge potential recall bias, inability to infer causality, and unmeasured cultural confounders (Page 19, Lines 466–472).

- Ethical considerations are well-addressed, but **more details on informed consent** during CDHS data collection would strengthen the manuscript’s transparency.

**Authors’ response:** We also elaborated on the informed consent process (Page 11, Lines 280–282).

## Minor Comments

- The abstract should explicitly state the *study design* (cross-sectional).
- The Abstract now explicitly mentions that this was a cross-sectional study.

**Authors’ response:** We have revised the manuscript accordingly; the study design was cross-sectional.

- Tables should be *formatted for readability*, particularly those with dense numerical data.

**Authors’ response:** Tables have been revised slightly for better readability.

- There are some *minor grammatical issues* a language edit would improve clarity.

**Authors’ response:** Minor grammatical edits were made by our senior research authors

- Improve variable categorization explanations* (e.g., why specific cutoff points for distance to healthcare were chosen).

**Authors’ response:** We followed the distance from home to the primary healthcare context in Cambodia based on Population coverage, road conditions, and distancing.

- Discuss dual-method contraceptive use (modern + traditional), if relevant.

**Authors’ response:** The CDHS does not specifically track dual-method use; modern and traditional methods are recorded separately.

## Recommendation

** Accept with Major Revisions and Minor Revisions** – The study is methodologically sound and contributes to public health knowledge, but revisions are needed to **clarify methodology, enhance result interpretation, and strengthen policy implications**.

I appreciate the authors' effort in conducting this research and look forward to seeing an improved version of the manuscript.

6. PLOS authors have the option to publish the peer review history of their article (what does this mean?). If published, this will include your full peer review and any attached files.

Do you want your identity to be public for this peer review? If you choose “no”, your identity will remain anonymous but your review may still be made public.

For information about this choice, including consent withdrawal, please see our Privacy Policy.

Reviewer #1: No

Reviewer #2: Yes: Betelihem Asrat Biru
